# Editorial: Eicosanoids in Cancer

**DOI:** 10.3389/fphar.2021.765214

**Published:** 2021-09-09

**Authors:** Nune Markosyan, Emer M. Smyth, Paola Patrignani, Emanuela Ricciotti

**Affiliations:** ^1^ Department of Medicine, Abramson Family Cancer Research Institute, Abramson Cancer Center, University of Pennsylvania, Philadelphia, PA, United States; ^2^ Department of Pathology and Cell Biology, Herbert Irving Comprehensive Cancer Center, Columbia University Medical Center, New York, NY, United States; ^3^ Department of Neuroscience, Imaging and Clinical Sciences, School of Medicine, CAST (Center for Advanced Studies and Technology), “G. d’Annunzio” University, Chieti, Italy; ^4^ Department of Systems Pharmacology and Translational Therapeutics, Perelman School of Medicine, Institute for Translational Medicine and Therapeutics, University of Pennsylvania, Philadelphia, PA, United States

**Keywords:** eicosanoids, cancer, arachidonic acid, oxylipins, inflammation

Eicosanoids, a family of bioactive lipid mediators discovered less than 100 years ago, have broad functions in homeostasis and in various physiological and pathological conditions. They are metabolites of arachidonic acid formed primarily through the action of cytosolic phospholipase A2-α. These bioactive lipids consist of three major groups of metabolites: prostaglandins (PG)s and thromboxane, formed by the cyclooxygenases (COX)s; leukotrienes (LT)s, lipoxins, and hydroxyeicosatetraenoic acids formed by the 5-lipoxygenase (LOX); epoxygenated fatty acids, formed by the cytochrome P450 (CYP). The importance of the eicosanoids was emphasized by the Nobel Prize, awarded in 1982, to Sune Bergström, Bengt Samuelsson and John Vane “for their discoveries concerning prostaglandins and related biologically active substances”. When produced on demand, these oxidized lipids are responsible for inflammation and its subsequent resolution with eventual return to pre-inflammation levels (Schmid and Brüne, 2021). However, unresolved, chronic inflammation, and pathogen/antigen persistence characterized by sustained PG and LT production and immune-suppression becomes a fertile soil for malignant transformation and tumor immune evasion. Based on these tumor-promoting features, inflammation was designated as an enabling characteristic among the hallmarks of cancer (Hanahan and Weinberg, 2011). As a consequence, eicosanoids became attractive targets in gastrointestinal (Wang and DuBois, 2018), colon (Guillem-Llobat et al., 2016; Patrignani et al., 2017), breast (Kundu et al., 2014; Markosyan et al., 2014), pancreatic (Markosyan et al., 2019), prostate, and lung (Hanaka et al., 2009) cancers, and in melanoma (Zelenay et al., 2015).

Eicosanoids in Cancer research topic combines 1) reviews presenting the current understanding of the role of COXs, (Johnson et al.; Smith et al.; Ching et al.), LOX (Tian et al.), and CYPs (Evangelista et al; Luo and Liu) pathway products in cancer, 2) articles presenting alternative regulators of eicosanoid pathways in cancer such as miR-574-5p (Emmerich et al.), nitro fatty acids (Piesche et al.), and extracellular ATP (Sharma et al.), 3) novel therapeutic approaches focusing on downstream targets in eicosanoid pathways and genes upregulated by COX-2 (Ching et al.; Hidalgo-Estevez et al.), as well as harnessing the potential of dietary manipulations to circumvent pro-tumorigenic effects of eicosanoids (Storniolo et al.; Luo and Liu).

A timely and thorough review by Johnson et al. focuses on the role of prostaglandins in the suppression of anti-tumor immunity through their paracrine effects on the immune cells in the tumor microenvironment. Given the revolution in cancer treatment afforded by immune checkpoint blockade and cellular immunotherapy, investigation into the mechanisms of resistance and sensitization to immunotherapy is becoming imperative. Data presented in the review suggests that blocking prostaglandin pathways can relieve immunosuppression or synergize with immunotherapies. Smith et al. discuss the role of all three groups of eicosanoids in gynecological malignancies and Ching et al. review the data on PGE_2_ receptors EP2 and EP4 signaling in cancer. The terminal prostanoid synthase inhibition and receptor antagonism are increasingly attractive approaches in cancer treatment as they are free of the dire side effects that have complicated the use of non-steroidal anti-inflammatory drugs and selective COX-2 inhibitors. EP4 antagonism emerges as a promising approach in cancer treatment, especially in combination with chemotherapy or checkpoint blockade.

As putative inflammatory mediators, elevated LTs are associated with tumor initiation, progression, neo-angio- and lymphogenesis, epithelial-to-mesenchymal transition, and metastasis. However, as discussed in the review by Tian et al., there is a body of evidence indicating that these lipid mediators may 1) alleviate immune-suppression through recruitment of CD8^+^ T cells, 2) maintain viable lymphatic vasculature, increasing the efficacy of immunotherapy. Similarly, several isoforms of CYPs are overexpressed in solid tumors, and due to their drug metabolizing capacity and pro-angiogenic/vasodilator functions, are implicated in chemoresistance, tumor growth and metastasis. Evangelista et al. review the contrasting roles of CYPs in physiological conditions and cancer and point out the difficulty of inhibition of these pathways in cancer due to their importance in non-cancer settings. Analyzing the pleotropic functions of CYPs in cancer, Luo et al. conclude that CYP- mediated eicosanoids derived from ω-3 fatty acids lack the pro-tumorigenic effects of their ω-6 derived counterparts and can be beneficial in cancer prevention and treatment. A study by Storniolo et al. furthers the notion of the possibility to alter the effect of eicosanoids in cancer by manipulating the composition of dietary fats, with high concentrations of ω-3 fatty acids inducing apoptosis of colorectal cancer cells and low concentrations being mitogenic for the same cell line.

A series of research articles within the Eicosanoids in Cancer research topic identify novel regulators of prostaglandin synthesis enzymes that can be targeted in inflammation as well as in cancer. Micro RNAs are more often post-transcriptional repressors of gene expression, but as shown by Emmerich et al., in human lung cancer cells, miR-574-5p induces PGE_2_ synthetic enzyme microsomal prostaglandin E synthase-1 (mPGES1) expression and promotes tumor growth. High expression of *PTGES*, the gene encoding mPGES1 protein, is associated with low CD8^+^ T cells infiltration and shorter patient survival (Kim et al., 2019), and a small molecular inhibitor of mPGES1 was shown to suppress tumor growth in a preclinical neuroblastoma mouse model (Kock et al., 2018). miR-574-5p emerges as a new target to control intra-tumoral mPGES1 expression, hence PGE_2_ levels, and tumor growth. Sharma et al. explore a possible mechanism of COX-2 upregulation following chemotherapy. In cervical, neuronal, and breast cancer cell lines, doxorubicin induced cell death results in increased release of ATP that coincides with up-regulation of COX-2 and metalloproteinases-2 and increased tumor cell invasion and migration. Further studies are needed to explore the potential of ATP-binding purinergic receptors as potential targets in cancer therapeutics. A review by Piesche et al. summarizes the data on nitro fatty acids (NFA) that can undergo Michael addition reactions and modify the function of target proteins such as Peroxisome Proliferator-Activated Receptor-γ, NF-κB, 5-LOX, and soluble epoxide hydrolase. This ability of NFAs can potentially be used to control inflammation and enhance cancer treatments. Finally, Hidalgo-Estevez et al. suggest that targeting genes regulated by COX-2 such as Dual Specificity Phosphatase 4 and 10, Programmed cell death 4, Trophoblast cell-surface antigen 2 and many from the Tumor growth factor β and p53 pathways can be a viable alternative to the use of COX-2 inhibitors in colon cancer.

Although widely studied as inflammatory mediators, the role of many eicosanoids in cancer has not been completely elucidated. PGE_2_ has justifiably received the most attention, but more research is needed to understand the contribution of other prostanoids, LTs, and CYP metabolites in cancer initiation, progression, spread, and treatment. Most importantly, anti-tumor therapies targeting these inflammatory pathways should be specifically tailored based on the tissue eicosanoid profile and receptor expression as well as their interplay with other cancer treatments.

## References

[B1] Guillem-Llobat P. Dovizio M. Bruno A. Ricciotti E. Cufino V. Sacco A. (2016). Aspirin Prevents Colorectal Cancer Metastasis in Mice by Splitting the Crosstalk between Platelets and Tumor Cells. Oncotarget 7 (22), 32462–32477. May 31. 10.18632/oncotarget.8655 27074574 PMC5078026

[B2] Hanahan D. Weinberg R. A. (2011). Hallmarks of Cancer: the Next Generation. Cell 144 (5), 646–674. Mar 4. 10.1016/j.cell.2011.02.013 21376230

[B3] Hanaka H. Pawelzik S. C. Johnsen J. I. Rakonjac M. Terawaki K. Rasmuson A. (2009). Microsomal Prostaglandin E Synthase 1 Determines Tumor Growth *In Vivo* of Prostate and Lung Cancer Cells. Proc. Natl. Acad. Sci. U S A. 106 (44), 18757–18762. Nov 3. 10.1073/pnas.0910218106 19846775 PMC2768589

[B4] Kim S. H. Roszik J. Cho S. N. Ogata D. Milton D. R. Peng W. (2019). The COX2 Effector Microsomal PGE2 Synthase 1 Is a Regulator of Immunosuppression in Cutaneous Melanoma. Clin. Cancer Res. 25 (5), 1650–1663. Mar 1. 10.1158/1078-0432.CCR-18-1163 30538110 PMC6397703

[B5] Kock A. Larsson K. Bergqvist F. Eissler N. Elfman L. H. M. Raouf J. (2018). Inhibition of Microsomal Prostaglandin E Synthase-1 in Cancer-Associated Fibroblasts Suppresses Neuroblastoma Tumor Growth. EBioMedicine 32, 84–92. Jun. 10.1016/j.ebiom.2018.05.008 29804818 PMC6021299

[B6] Kundu N. Ma X. Kochel T. Goloubeva O. Staats P. Thompson K. (2014). Prostaglandin E Receptor EP4 Is a Therapeutic Target in Breast Cancer Cells with Stem-like Properties. Breast Cancer Res. Treat. 143 (1), 19–31. Jan. 10.1007/s10549-013-2779-4 24281828 PMC3889836

[B7] Markosyan N. Chen E. P. Smyth E. M. (2014). Targeting COX-2 Abrogates Mammary Tumorigenesis: Breaking Cancer-Associated Suppression of Immunosurveillance. Oncoimmunology 3, e29287. Jun 30. 10.4161/onci.29287 25114833 PMC4126836

[B8] Markosyan N. Li J. Sun Y. H. Richman L. P. Lin J. H. Yan F. (2019). Tumor Cell-Intrinsic EPHA2 Suppresses Anti-tumor Immunity by Regulating PTGS2 (COX-2). J. Clin. Invest. 129 (9), 3594–3609. Jun 4. 10.1172/JCI127755 31162144 PMC6715369

[B9] Patrignani P. Sacco A. Sostres C. Bruno A. Dovizio M. Piazuelo E. (2017). Low-Dose Aspirin Acetylates Cyclooxygenase-1 in Human Colorectal Mucosa: Implications for the Chemoprevention of Colorectal Cancer. Clin. Pharmacol. Ther. 102 (1), 52–61. Jul. 10.1002/cpt.639 28139830

[B10] Schmid T. Brüne B. (2021). Prostanoids and Resolution of Inflammation - beyond the Lipid-Mediator Class Switch. Front. Immunol. 12, 714042. Jul 12. 10.3389/fimmu.2021.714042 34322137 PMC8312722

[B11] Wang D. DuBois R. N. (2018). Role of Prostanoids in Gastrointestinal Cancer. J. Clin. Invest. 128 (7), 2732–2742. Jul2. 10.1172/JCI97953 29733297 PMC6026007

[B12] Zelenay S. van der Veen A. G. Böttcher J. P. Snelgrove K. J. Rogers N. Acton S. E. (2015). Cyclooxygenase-Dependent Tumor Growth through Evasion of Immunity. Cell 162 (6), 1257–1270. Sep 10. 10.1016/j.cell.2015.08.015 26343581 PMC4597191

